# An Adaptive Framework for Remaining Useful Life Prediction Integrating Attention Mechanism and Deep Reinforcement Learning

**DOI:** 10.3390/s25206354

**Published:** 2025-10-14

**Authors:** Yanhui Bai, Jiajia Du, Honghui Li, Xintao Bao, Linjun Li, Chun Zhang, Jiahe Yan, Renliang Wang, Yi Xu

**Affiliations:** 1 School of Computer Science and Technology, Beijing Jiaotong University, Beijing100044, China; baiyh@bjtu.edu.cn (Y.B.); 22281062@bjtu.edu.cn (J.D.); 22281119@bjtu.edu.cn (X.B.); chzhang1@bjtu.edu.cn (C.Z.); 20112038@bjtu.edu.cn (J.Y.); 21111099@bjtu.edu.cn (R.W.); 23111101@bjtu.edu.cn (Y.X.); 2 Engineering Research Center of Network Management Technology for High Speed Railway, Ministry of Education, Beijing 100816, China; 3 China National Energy Co., Ltd., Beijing 100048, China; 11591129@ceic.com

**Keywords:** remaining useful life (RUL) prediction, attention mechanism, Deep Deterministic Policy Gradient (DDPG), Functional Alignment Resampling (FAR)

## Abstract

The prediction of Remaining Useful Life (RUL) constitutes a vital aspect of Prognostics and Health Management (PHM), providing capabilities for the assessment of mechanical component health status and prediction of failure instances. Recent studies on feature extraction, time-series modeling, and multi-task learning have shown remarkable advancements. However, most deep learning (DL) techniques predominantly focus on unimodal data or static feature extraction techniques, resulting in a lack of RUL prediction methods that can effectively capture the individual differences among heterogeneous sensors and failure modes under complex operational conditions. To overcome these limitations, an adaptive RUL prediction framework named ADAPT-RULNet is proposed for mechanical components, integrating the feature extraction capabilities of attention-enhanced deep learning (DL) and the decision-making abilities of deep reinforcement learning (DRL) to achieve end-to-end optimization from raw data to accurate RUL prediction. Initially, Functional Alignment Resampling (FAR) is employed to generate high-quality functional signals; then, attention-enhanced Dynamic Time Warping (DTW) is leveraged to obtain individual degradation stages. Subsequently, an attention-enhanced of hybrid multi-scale RUL prediction network is constructed to extract both local and global features from multi-format data. Furthermore, the network achieves optimal feature representation by adaptively fusing multi-source features through Bayesian methods. Finally, we innovatively introduce a Deep Deterministic Policy Gradient (DDPG) strategy from DRL to adaptively optimize key parameters in the construction of individual degradation stages and achieve a global balance between model complexity and prediction accuracy. The proposed model was evaluated on aircraft engines and railway freight car wheels. The results indicate that it achieves a lower average Root Mean Square Error (RMSE) and higher accuracy in comparison with current approaches. Moreover, the method shows strong potential for improving prediction accuracy and robustness in varied industrial applications.

## 1. Introduction

With the rapid digital and intelligent transformation of manufacturing, key equipment components are increasingly evolving toward greater scale and integration [1]. When mechanical equipment components operate under complex conditions, degradation rates and degradation patterns exhibit significant individual variability. Based on daily inspection and regular maintenance, planned preventive maintenance in traditional maintenance strategies results in serious delays in fault monitoring and weak preventive abilities [2]. The development and application of Prognostics and Health Management (PHM) technology, which relies on the real-time health status of equipment, are dedicated to avoiding excessive maintenance, reducing false-alarm rates, and ensuring safe equipment operation by dynamically scheduling maintenance. As an essential aspect of PHM, Remaining Useful Life (RUL) prediction focuses on continuously monitoring equipment conditions and predicting failure times to extend the operational duration of the equipment and improve economic efficiency. Typical RUL prediction approaches are generally categorized into physics-based approaches and data-driven approaches [3].

Physics-based approaches represent the degradation process by establishing physical models of a complex system. For instance, Zhang et al. [4] developed a capacity-cycling degradation model to estimate the RUL of online lithium-ion batteries. In their study, they estimated the core temperature, state of charge, and battery capacity by leveraging thermal and Coulomb SOC models. Similarly, Shutin et al. [5] proposed a degradation model integrating tribological theory with the physical wear mechanisms of rolling bearings to predict the RUL of hydrodynamic bearings. In another study, Protopapadakis et al. [6] implemented an understandable AI-assisted RUL estimation method for turbine engines by leveraging a degradation model derived from aerothermodynamics and analyzing measurement data. Although physics-based methods are easily interpretable, their reliance on in-depth information about the principle of equipment failure mechanisms and domain-specific knowledge limits their generalization capability for unknown complex systems.

The widespread adoption of intelligent sensors and advances in big data technology are rapidly increasing the volume of monitoring data available to industries. Extracting value from these multi-source, heterogeneous datasets enhances Remaining Useful Life (RUL) prediction. Thus, data-driven RUL prediction is becoming popular across industrial and academic domains. Currently, data-driven remaining useful life prediction mainly utilizes three prevalent approaches: statistical methods, machine learning (ML), and deep learning (DL). Statistical methods such as Hidden Markov Models (HMMs), Kalman filters, and Wiener processes analyze statistical distributions, trends, and patterns in historical data to model equipment degradation and predict the RUL. Zhang et al. [7] successfully addressed challenges related to nonlinearity, state transitions, and stochasticity in predicting the remaining useful life of lithium-ion batteries. Their approach combines a nonlinear drift-driven Wiener process, a Markov chain-switching model, and a fuzzy system. Furthermore, they significantly improved the model’s reliability in terms of predictive precision by introducing adaptive filtering techniques in dynamic environments. Although statistical models are computationally efficient, they have limited ability in modeling nonlinear relationships. As a result, they are frequently combined with ML approaches, including Support Vector Machines (SVMs), Random Forests (RFs), and decision trees, to derive valuable information from the Probability Density Functions (PDFs) of datasets [8]. For instance, Alfarizi et al. [9] constructed a two-stage model to forecast the remaining useful life of experimental bearings. In the first stage, the input signals were decomposed into different frequency bands using empirical mode decomposition, eliminating irrelevant frequencies and highlighting fault characteristics. In the second stage, they combined a random forest model with Bayesian hyperparameter tuning to enhance the accuracy of RUL prediction. Although ML methods show strong nonlinear modeling capabilities, the abovementioned component-level RUL prediction approaches grounded in ML methodologies demand an index that reflects degradation levels and heavily rely on feature engineering, rendering them unsuitable for RUL prediction using multidimensional time-series data (MTSD).

As a subfield of ML, DL provides substantial technological advantages for component RUL prediction, owing to its strengths in modeling intricate nonlinear relationships, processing high-dimensional data, and automating feature engineering. Recurrent Neural Network (RNNs), which are capable of capturing temporal degradation patterns, are extensively used for estimating RUL [10]. Nevertheless, for extended time-series data, they are prone to issues such as gradient explosion and reduced computational efficiency, limiting their practical deployment in industrial settings [11]. As enhanced successors of RNNs, the use of Long Short-Term Memory (LSTM) and Gated Recurrent Unit (GRUs) strengthens the ability to model long-term dependencies and mitigate gradient vanishing and explosion via gating mechanisms and gradient optimization strategies, establishing them as dominant approaches in remaining useful life estimation. Chui et al. [12] presented a Non-dominated Sorting Genetic Algorithm II (NSGA-II)-based RUL prediction model that combines the short-term prediction strengths of RNNs with the long-term prediction capabilities of LSTM, ultimately efficiently addressing challenges related to machine downtime and redundant maintenance during the running to failure and preventive maintenance of turbofan engines. Beyond long-term temporal dependencies, it is essential to consider the local features of MTSD. To combine global temporal patterns and local and global information for the RUL prediction of equipment, Cao et al. [13] proposed a parallel RUL prediction architecture combining a multi-scale CNN (MSCNN) and multi-scale LSTM (MSLSTM) to extract multi-dimensional health indicators, effectively reducing the local fluctuation caused by the CNN. Then, they used a dynamic time warping (DTW)-based similarity-matching algorithm to identify historical training samples with degradation trends similar to the test sequence. Finally, they achieved accurate prediction for railway freight car wheels. Duan et al. [14] treated mechanical monitoring data as “natural language sequences of machines” and input them into a Transformer. This methods begins by utilizing its attention layers to highlight core time-step information and compute the output in parallel, followed by the integration of two attention mechanisms in the Transformer structure with LSTM in the encoder to extract both local and long-term temporal dependency information of the degradation process. Finally, it employs a nonlinear Wiener process (NWP) to calculate the PDF of the RUL. Song et al. [15] addressed the impact of prediction outcomes of the complex interactions among high-dimensional variables in MTSD by constructing multi-dimensional feature-correlated spatiotemporal (MFCST) graphs to implement feature extraction for data in different formats while employing a stacked long short-term memory (ST-LSTM) network to comprehensively explore local and global temporal pattern of MTSD. Then, they strategically weighted spatial and temporal patterns to enhance the model’s generalization ability and spatial perception of high-dimensional variable feature structures. Currently, DL-based methods for RUL prediction frequently adopt averaging or predefined weights to address issues such as high-frequency noise, local fluctuations, and missing values in high-frequency sensor data. Additionally, models trained using randomly sampled training data cannot adequately leverage the intrinsic correlations among sensors and their different levels of contributionsto the degradation process, which limits the model’s predictive capability on real-time data. To resolve these difficulties, regularization techniques are typically used, but they require manual parameter tuning, increasing the cost of human intervention.

In contrast, deep reinforcement learning (DRL) stands out as an ML technique, providing significant support for the capture of temporal dependencies in equipment RUL prediction due to its remarkable ability to learn optimal strategies through interaction with the environment, achieving stronger exploration and generalization capabilities [16]. In particular, DRL is divided into two approaches: the value-based Deep Q-Network (DQN) algorithm and the Deterministic Policy Gradient (DPG) algorithm. DQN uses deep neural networks to approximate the Q-value function and improves stability through experience replay and target networks. On this basis, Yao et al. [17] proposed a Deep Transfer Reinforcement Learning (DTRL) network based on LSTM, which utilizes novel Q-function updates and transfer strategies to estimate the RUL of machinery operating under similar tool and cutting conditions. DQN demonstrates exceptional performance in discrete action spaces; however, it exhibits significant limitations when applied to continuous action spaces. In contrast, DPG directly optimizes policy parameters through gradient ascent to maximize expected rewards. Despite its theoretical elegance, DPG lacks scalability in deep learning frameworks [18]. To address this, Actor–Critic algorithm was developed as an enhancement of DPG, combining value-function approximation with policy gradient methods. By leveraging the advantage function, it effectively reduces variance in gradient estimation [19]. Nevertheless, the Actor–Critic model’s training remains unstable due to high variance. Building upon this, the Deep Deterministic Policy Gradient (DDPG) algorithm extends the Actor–Critic strategy and is specifically designed for continuous action spaces. DDPG incorporates deterministic policies and target networks, successfully scaling DPG to high-dimensional, continuous action spaces and improving training stability. Zheng et al. [20] designed a DRL model trained based on the Twin Delayed DDPG (TD3) algorithm. The model leverages the powerful feature representation of DL while maintaining temporal dependencies across samples through RL, enabling accurate RUL estimation for rolling bearings. Hu et al. [21] integrated DRL with a Markov Decision Process (MDP) framework to learn to derive an optimal strategy for RUL prediction. Although current deep fusion techniques mitigate the shortcomings of conventional DL in RUL prediction and improve model robustness and adaptability, the use of DRL to dynamically adjust time-scale parameters and prioritize critical degradation phases remains an unresolved challenge.

To overcome the above limitations, this study presents ADAPT-RULNet, an adaptive RUL prediction framework that integrates attention mechanisms with DRL based on a hybrid network. The proposed framework enhances the quality of input signals by utilizing Functional Alignment Resampling (FAR) for MTSD preprocessing and using DTW to construct personalized datasets with similar degradation stages. The framework extracts both local and long-term degraded information from MTSD through an attention-enhanced multi-scale CNN and LSTM. These features are then integrated through an adaptive Bayesian fusion layer, thereby achieving better prediction performance. Furthermore, the framework introduces the DDPG to adaptively adjust critical time-scale parameters to obtain an optimal global balance between prediction accuracy and model complexity. To validate the effectiveness of ADAPT-RULNet, extensive experiments were carried out on two datasets in comparison with the advanced method. The experimental results suggest that ADAPT-RULNet outperforms all other approaches, on average.

The main contributions of this work are outlined below:

1. We propose a robust and precise data preprocessing framework for mechanical equipment RUL prediction. By employing the novel FAR method for data signal optimization, we effectively address the issues of noise, heterogeneity, and inconsistent time-series lengths in raw sensor data, thereby providing high-quality input signals for subsequent feature extraction. Furthermore, leveraging the attention-based DTW-enhanced model leads to the selection of degradation stages with highly similar processes across devices to construct a personalized dataset with high-quality and consistent degradation.
2. We construct an attention-enhanced multi-scale parallel feature extraction model. The proposed method leverages a multi-scale CNN with spatial–temporal attention mechanisms to extract temporal features and local degradation patterns from multi-modal sensor data. Simultaneously, multi-scale LSTM with multi-head attention mechanisms is employed to capture multi-modal temporal features and global degradation patterns. The multi-dimensional features are adaptively fused using Bayesian probability to enhance the accuracy of RUL prediction.
3. We introduce a complexity–efficiency balancing strategy based on DDPG. This approach reformulates the parameter optimization process in RUL prediction as an MDP. This strategy leverage the experience replay mechanism and target-network soft update technique within the DDPG framework to adaptively optimize the time-window size and the number of similar samples of key parameters during the construction of the feature extraction dataset. This ensures a global balance between prediction performance and model complexity.
4. With respect to practical application prospects, the approach was validated on public and industrial datasets, confirming its effectiveness. The prediction results surpass those of existing CNN-LSTM models, demonstrating strong potential for intelligent maintenance in real industrial applications.

The structure of this study is outlined as follows: Section 2 reviews the related research and technical methodologies. Section 3 describes the proposed adaptive RUL prediction framework. Section 4 analyzes the experimental results, while Section 5 discusses the conclusions and outlines future research directions.

## 2. Theoretical Basis

### 2.1. Multi-Head Self-Attention Mechanism

**Figure 1:**
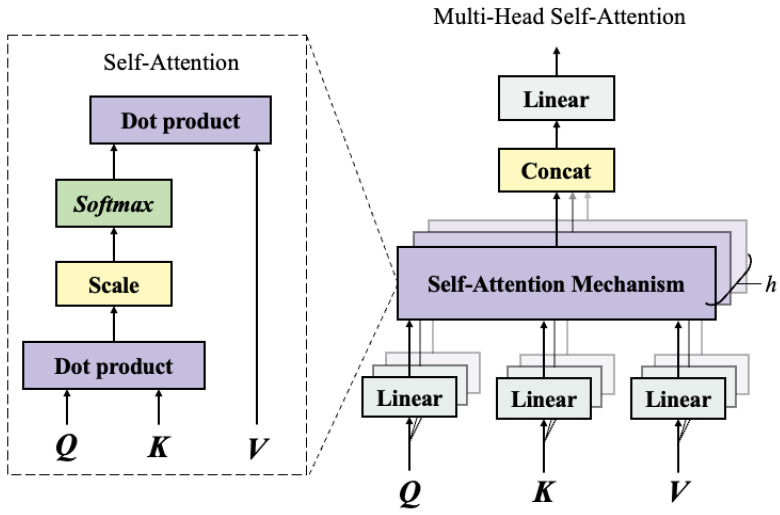
The architecture of the multi-head self-attention mechanism.

The attention mechanism is inspired by the human visual cognitive process. Its fundamental principle is to rapidly scan the global image, identify and focus on the target regions that require special attention, and allocate more attentional resources to these regions, thereby obtaining richer detailed information and suppressing irrelevant interference. The self-attention mechanism is a specialized form of the attention mechanism primarily used to capture dependencies within the same sequence. However, a single attention head often fails to fully capture the diverse features in the input data. To improve the expressive power of their model, Vaswani et al. [22] proposed the Multi-Head Self-Attention (MHSA) mechanism, as represented in Figure 1.

The MHSA mechanism combines multiple scaled dot-product attention modules in parallel, allowing different “heads” to attend to distinct portions of the input data. Specifically, each self-attention module independently computes its output, and these outputs are integrated through a weighted summation to produce the final result of the MHSA mechanism. This design not only enables the capture of more diverse features and patterns but also significantly reduces computational costs due to its parallel computation characteristics. Compared to a single self-attention mechanism, the MHSA mechanism can more stably capture complex dependencies and structural information while demonstrating stronger robustness.

In the MHSA mechanism, the input data is first transformed to generate the query (Q), key (K), and value (V) vectors through distinct linear projections. Subsequently, each attention head independently computes its corresponding attention weights and produces the output of that head via a weighted summation process. Finally, the outputs from all attention heads are concatenated and passed through a final linear transformation. The combined result is expressed in Equation (1).

(1) $$\mathrm{MultiHead}\left(Q,K,V\right)=\mathrm{Concat}\left(h_{1},\ldots ,h_{H}\right)W^{O}$$

where $h_{i}$ denotes the output of a single head, as shown in Equation (2):

(2) $$\begin{array}{cc} h_{i} & =\mathrm{Attention}\left(Q_{i},K_{i},V_{i}\right)=\mathrm{softmax}\;\left(\frac{Q_{i}K_{i}^{T}}{\sqrt{d_{k}}}\right)V_{i},\;i=1,2,\ldots ,H\\ Q_{i} & =XW_{q}^{i},\;K_{i}=XW_{k}^{i},\;V_{i}=XW_{v}^{i} \end{array}$$

where $W_{q}^{i}$, $W_{k}^{i}$, $W_{v}^{i}$, and $W^{O}$ denote learnable weight matrices; Concat(·) represents the concatenation operation; and H signifies the number of self-attention heads. The attention distribution coefficient matrix is computed based on the query matrix (Q) and the key matrix (K) and is subsequently normalized using the softmax function applied to the scaled dot-product results.

### 2.2. Convolutional Neural Networks

Convolutional Neural Networks (CNNs) were first proposed by LeCun et al. in 1989 [23], representing a specialized class of feedforward neural networks inspired by the biological visual system. By emulating three structural properties of visual cortex cells—local receptive fields, shared weights, and subsampling—CNNs preserve invariance to translation, scaling, and distortion, thereby enabling the extraction of topological features from raw input data. Additionally, a CNN can directly process 2D array inputs, avoiding the complex feature extraction and data reconstruction steps typically required by traditional recognition algorithms.

The convolutional layer consists of convolution and activation operations, performing convolution on the local receptive field using a filter (also referred to as a weight) window. Because of the weight-sharing property inherent in the convolutional layers of a CNN, the model complexity is significantly reduced, while the risk of overfitting caused by an excessive number of network parameters is effectively mitigated [24]. Given a 2D input (X) to the convolutional layer of a CNN, the output is computed as shown in Equation (3):

(3) $$C_{n}=\sigma \;\left(\sum_{k=1}^{K}W_{n,k}⊗X_{k}+b_{n}\right)$$

where K represents the input-channel count and *n* denotes the number of convolutional kernels. $C_{n}$ signifies the *n*-th hidden feature. $W_{n,k}$ corresponds to the weight parameters between the *n*-th convolutional kernel and the *k*-th input channel, while $b_{n}$ represents the bias term associated with the *n*-th convolutional kernel. The σ(·) function denotes the function of activation, and ⊗ symbolizes the convolution operation.

The pooling layer, also known as the downsampling layer, primarily serves to introduce translation invariance, reduce the dimensionality of the feature maps output by the convolutional layer, mitigate the risk of overfitting, and decrease computational complexity. Using degradation data of critical mechanical equipment as an example, determining whether a component has reached its end-of-life failure is more critical than identifying which specific part of the data represents the degradation phase. Common pooling operations include max pooling and average pooling. In this study, max pooling is employed, which extracts the maximum value within a rectangular neighborhood, as shown in Equation (4):

(4) $$P\left(i,j\right)=\max_{(x,y)\in R}C\left(x,y\right)$$

where (x,y) denotes the size of the pooling window, P(i,j) represents the output feature value after pooling, and maxC(·) indicates the maximum value within the pooling region.

### 2.3. Attention-Enhanced Depthwise Separable Convolution

Depthwise Separable Convolution (DSC) [25] enhances model efficiency by decomposing standard convolution into depthwise convolution and pointwise convolution, which collectively generate the final feature map. Depthwise convolution operates independently on each input channel, and its expression is shown in Equation (5):

(5) $$\mathrm{Depthwise}\mathrm{Conv}:\;Y_{i,j,k}=\sum_{m,n}K_{m,n,k}\cdot X_{i+m,\;j+n,\;k}$$

On the other hand, pointwise convolution integrates channel information through a 1×1 convolutional kernel, as shown in Equation (6):

(6) $$\mathrm{Pointwise}\mathrm{Conv}:\;Z_{i,j,l}=\sum_{k}W_{k,l}\cdot Y_{i,j,k}$$

where X represents the input feature map, K denotes the depthwise convolution kernel, W is the pointwise convolution kernel, Y corresponds to the output of the depthwise convolution, and Z signifies the output of the pointwise convolution.

To augment the model’s capability in identifying crucial features, a Channel Attention Module (CAM) and Spatial Attention Module (SAM) are integrated. Specifically, the CAM dynamically assigns weights to each channel through a combination of Global Average Pooling (GAP) and fully connected layers, thereby emphasizing the channel features that contribute most significantly to the task, as shown in Equation (7):

(7) $$M_{c}=\sigma \;\left(W_{1}\left(W_{0}\left(\mathrm{GAP}\left(X\right)\right)\right)+b_{1}\right)$$

where GAP(X) denotes the global average pooling operation, $W_{0}$ and $W_{1}$ denote the weights of the fully connected layers, $b_{1}$ represents the bias, and σ signifies the Sigmoid activation function.

The SAM dynamically allocates weights to each spatial location by concatenating max pooling and average pooling results, followed by a convolutional operation, thereby emphasizing the spatial regions that contribute most significantly to the task, as shown in Equation (8):

(8) $$M_{s}=\sigma \;\left({\mathrm{Conv}}_{c\times c}\left([\mathrm{MaxPool}(X);\mathrm{AvgPool}(X)]\right)\right)$$

where ${\mathrm{Conv}}_{c\times c}$ denotes the c×c convolutional operation and [MaxPool(X);AvgPool(X)] represents the concatenation of the results from max pooling and average pooling. The weighting expression for channel attention is shown in (9):

(9) $$X_{att}=M_{c}•M_{s}•X$$

where $X_{att}$ represents the feature map after attention weighting.

### 2.4. LSTM Network

**Figure 2:**
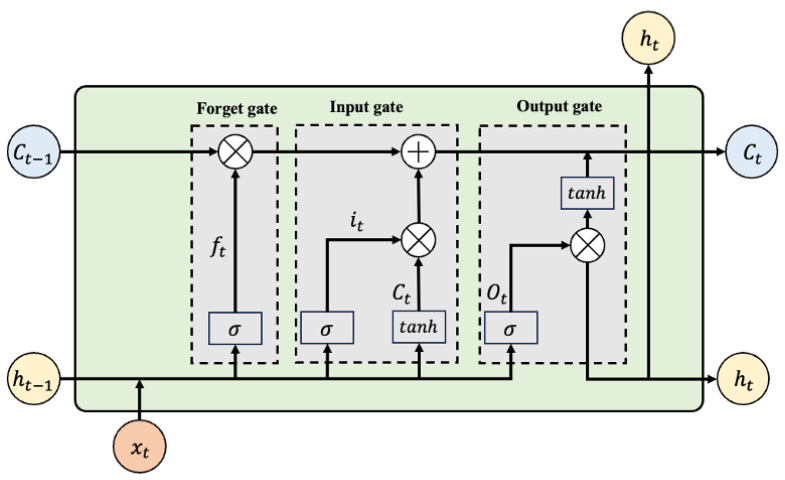
Structure of the LSTM network.

The LSTM network [26] is a specialized variant of the Recurrent Neural Network (RNN). By incorporating a gating mechanism, the LSTM network dynamically adjusts the memory state. This mechanism facilitates the effective transfer of information from previous time steps to subsequent units, resolving the issue of long-term dependencies in sequential data. Furthermore, LSTM effectively mitigates the common problems of vanishing and exploding gradients in RNNs, providing robust support for the modeling of long sequential data. As shown in Figure 2, the framework of LSTM primarily consists of three gating units: the forget gate ($f_{t}$), the input gate ($i_{t}$), and the output gate ($o_{t}$).

### 2.5. Deep Reinforcement Learning

DRL is a theoretical framework grounded in the MDP. Combining the representational power of DL and the decision-making mechanisms of RL, DRL greatly improves the learning and decision-making abilities of agents in high-dimensional state spaces.

The underlying mechanism of DRL involves persistent interaction cycles between agents and environments. Based on observed environmental states, agents choose and implement actions, obtaining subsequent environmental responses in reward or penalty form. Through continuous optimization of its policy, the agent gradually learns to make optimal decisions in a given environment, aiming to maximize the cumulative reward [27]. The action–value function of DRL is expressed in Equation (10):

(10) $$Q^{\pi }\left(s,a\right)=E_{\pi }\left[\sum_{t=0}^{\infty }\gamma^{t}r_{t+k}\;|\;s_{k}=s,a_{k}=a\right],\;s\in S,\;a\in A,\;r\in \left(0,1\right)$$

where S denotes a finite set of states and A represents a finite set of actions. $Q^{\pi }\left(s,a\right)$ is the action–value function, which represents the expected discounted cumulative reward obtained by selecting action a in state s and subsequently following policy π. Here, $E_{\pi }\left[\cdot \right]$ denotes the trajectories generated by policy π, $\gamma^{t}$ is the discount factor applied to the reward received *t* steps in the future, and $r_{t+k}$ represents the immediate reward received at time step k+t.

**Figure 3:**
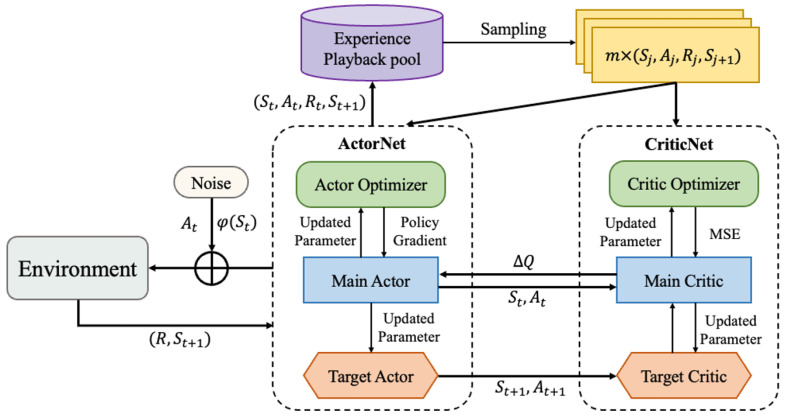
The network architecture of the DDPG algorithm.

The Deep Deterministic Policy Gradient (DDPG) method adopted in this study is an extension of the Actor–Critic framework specifically designed for continuous action spaces. The network architecture of DDPG primarily consists of three modules: the actor network (policy network), the critic network (value network), and the target network (employed to stabilize training). The detailed architecture and interaction logic of DDPG are illustrated in Figure 3.

The actor network is tasked with learning a deterministic policy, directly generating actions, while the critic network evaluates the value (Q-value) of the actions produced by the actor. At each time step (*t*), the agent selects a deterministic action ($a_{t}$) based on the current state ($s_{t}$) through its actor network. Upon execution of this action, the environment computes the corresponding reward ($r_{t}$) based on performance metrics and provides the subsequent state ($s_{t+1}$). The agent then stores the transition ($\left(s_{t},a_{t},r_{t},s_{t+1}\right)$) in the experience replay buffer. Once the buffer accumulates a sufficient amount of experience, the agent randomly samples a batch of data to update the parameters of both the actor and critic networks.

The critic network is updated by minimizing the mean squared error between the target Q-value and the current Q-value. The target value is defined in Equation (11):

(11) $$y_{i}=r_{i}+\gamma \;Q^{\prime }\left(s_{i+1},\mu^{\prime }\left(s_{i+1};\varphi^{\prime }\right);\theta^{\prime }\right)$$

where θ denotes the parameters of the critic network, which are optimized to learn the Q-value function (Q(s,a;θ)). Simultaneously, $\theta^{\prime }$ represents the parameters of the target critic network used to compute the target Q-value. The critic network minimizes the loss (L(θ)) through Equation (12):

(12) $$L\left(\theta \right)=\frac{1}{N}\sum_{i=1}^{N}{\left(y_{i}-Q\left(s_{i},a_{i};\theta \right)\right)}^{2}$$

Meanwhile, the actor network updates its parameters (φ) via the policy gradient, as shown in Equation (13):

(13) $$\nabla_{\varphi }J\left(\varphi \right)\approx \frac{1}{N}\sum_{i=1}^{N}\left(\nabla_{a}Q\left(s_{i},a;\theta \right){|}_{a=\mu (s_{i};\varphi )}\cdot \nabla_{\varphi }\mu \left(s_{i};\varphi \right)\right)$$

To prevent training instability caused by abrupt changes in the target network parameters, a “soft update” strategy is implemented, as formalized in Equation (14):

(14) $$\begin{array}{cccc} \varphi^{\prime } & \leftarrow \tau \varphi +\left(1-\tau \right)\varphi^{\prime }\; & \; & (\mathrm{update}\;\mathrm{target}\;\mathrm{Actor})\\ \theta^{\prime } & \leftarrow \tau \theta +\left(1-\tau \right)\theta^{\prime }\; & \; & (\mathrm{update}\;\mathrm{target}\;\mathrm{Critic}) \end{array}$$

where τ represents the soft update coefficient, which governs the synchronization rate of the target network parameters towards the primary network.

## 3. Proposed Method

**Figure 4:**
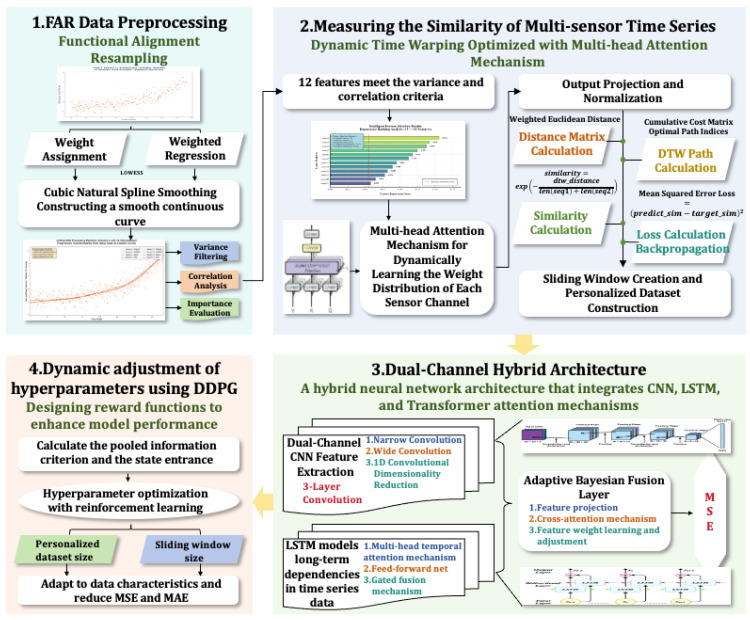
Framework of the proposed ADAPT-RULNet.

To efficiently integrate heterogeneous sensor data, mine the degradation patterns of mechanical equipment components under complex operating conditions, and accurately predict the RUL, this paper proposes an adaptive RUL prediction framework—ADAPT-RULNet, which integrates attention mechanisms and deep reinforcement learning, as illustrated in Figure 4.

The proposed framework consists of four tightly coupled modules:

1. **Data preprocessing** employs Functional Alignment Resampling (FAR) to optimize raw sensor signals by addressing noise, heterogeneity, and inconsistent time-series lengths.
2. **Personalized dataset construction** utilizes attention-enhanced Dynamic Time Warping (DTW) to build similarity-based degradation stages, ensuring that samples with highly similar degradation trajectories are grouped together.
3. **Hybrid network-based RUL prediction** constructs a hybrid architecture combining multi-scale CNN (MSCNN) and multi-scale LSTM (MSLSTM) with attention mechanisms and applies Bayesian fusion for adaptive feature integration.
4. **Reinforcement learning-based adaptive parameter tuning** introduces a Deep Deterministic Policy Gradient (DDPG) to adaptively adjust critical parameters such as time-window size and the number of selected similar samples, balancing prediction accuracy with model complexity.

These modules work synergistically to remarkably enhance the model’s robustness and generalization capability under complex operating conditions.

### 3.1. Data Preprocessing

In real-world industrial scenarios, the operational conditions of mechanical equipment exhibit high complexity, and their degradation processes demonstrate significant individual variability. Data collected from multi-source sensors often suffer from missing values, nonlinear characteristics, high levels of noise interference, unequal-length time series, and multi-source heterogeneity. Traditional data preprocessing methods encounter difficulty in capturing intricate degradation patterns. To tackle these issues, this paper introduces the Functional Adaptive Regression (FAR) approach, which reconstructs temporal continuity to better capture local or global degradation trends. By transforming time-series signals into functional signals, it facilitates the subsequent RUL prediction process.

Locally Weighted Scatterplot Smoothing (LOWESS) [28] and Cubic Natural Spline (CNS) smoothing serve as the core methodologies of FAR, enabling the transformation of raw time-series signals into functional signals. LOWESS employs localized smoothing techniques to effectively capture high-frequency noise and local fluctuations of the MTSD to adapt the local variations in the time series. Let $X=x{\left(i,u\right)}_{t}$, where $t=\{t_{1},t_{2},\ldots ,t_{n}\}$ represents the duration of the time series, $x=\{x_{1},x_{2},\ldots ,x_{m}\}$ represents the number of devices, and $u=\{u_{1},u_{2},\ldots ,u_{d}\}$ signifies the number of sensors. The term $x(\bar{i},u)$ represents the average value of all $x{\left(i,u\right)}_{t}$ within a local window (*h*), as illustrated in Equation (15):

(15) $$x(\bar{i},u)=\frac{1}{N}\sum_{j\in h}x{\left(i,u\right)}_{j}$$

The smoothed value ($\hat{x}{\left(i,u\right)}_{t}$) of LOWESS is computed through locally weighted regression, as shown in Equation (16):

(16) $$\hat{x}{\left(i,u\right)}_{t}=\beta_{0}+\beta_{1}\left(x{\left(i,u\right)}_{t}-x(\bar{i},u)\right)$$

where $\beta_{0}$ and $\beta_{1}$ are the regression coefficients obtained through weighted least squares and the weights ($\omega_{ij}$) are defined as shown in Equation (17):

(17) $$\begin{array}{c} \omega_{ij}={\left(1-{\left|\frac{x{\left(i,u\right)}_{j}-x{\left(i,u\right)}_{t}}{h}\right|}^{3}\right)}^{3},\;\left|x{\left(i,u\right)}_{j}-x{\left(i,u\right)}_{t}\right|\ll h\\ \min_{\beta_{0},\beta_{1}}\sum_{j}\omega_{ij}{\left(x_{j}^{\left(i,u\right)}-\left(\beta_{0}+\beta_{1}\left(x_{j}^{\left(i,u\right)}-x_{t}^{\left(i,u\right)}\right)\right)\right)}^{2} \end{array}$$

where $x{\left(i,u\right)}_{j}$ is the observed value of time-series signal $x_{I}$ at time step *j*.

The CNS interpolation method is applied to the multi-channel functional signals ($\hat{x}{\left(i,u\right)}_{t}$) to perform global fitting, generating continuous and smooth functional signal data. The fitting function ($g_{m}\left(X\right)$) is given by Equation (18):

(18) $$g_{m}\left(X\right)=a_{m}+b_{m}\left(X-X_{m}\right)+c_{m}{\left(X-X_{m}\right)}^{2}+d_{m}{\left(X-X_{m}\right)}^{3}$$

where coefficients $a_{m}$, $b_{m}$, $c_{m}$, and $d_{m}$ are obtained by minimizing the following objective function:

(19) $$\min_{\beta_{m}}\sum_{i=1}^{n}{\left(\hat{x}{\left(i,u\right)}_{t}-g_{m}\left(X\right)\right)}^{2}+\lambda \int {\left(g_{m}^{\prime \prime }\left(X\right)\right)}^{2}\;dX$$

where λ is the smoothing parameter, controlling the smoothness of the fitted curve, and $g_{m}^{\prime \prime }\left(X\right)$ is the second derivative of the spline function, used to measure the curvature of the curve. Finally, by performing global fitting on the smoothed data, a continuous functional signal ($X_{i}\left(t\right))$ is generated, as shown in Equation (20):

(20) $$X\left(t\right)=\sum_{m=1}^{M}\beta_{m}\;h_{m}\left(X\right)$$

### 3.2. Construction of Personalized Datasets with Similarity Degradation Stages

To completely capture the local suddenness and long-term trends of degradation behavior in mechanical equipment throughout the entire life cycle, this paper integrates a multi-head attention mechanism with DTW for personalized dataset construction (Attention-DTW). This approach overcomes the limitation of traditional DTW in terms of ignoring the differences among various sensors during the degradation process due to fixed weights. At the same time, it addresses the limitation of traditional Euclidean distance in effectively measuring the waveform similarity between two time series. By selecting the most relevant historical degradation samples for the test sequence, this method better captures the time-varying characteristics in dynamic uncertain environments and identifies similarities among operational signals, thereby providing reliable dataset support for the accuracy and robustness of RUL prediction.

Specifically, let the input MTSD be $X=\{x_{1},x_{2},\ldots ,x_{U}\}$, where $x_{u}\in R^{T}$ represents the time-series data of the *u*-th sensor. For the test sequence ($X_{v}$) and the historical sequence ($X_{i}$), the attention scores of each sensor channel are first calculated, as shown in Equation (21):

(21) $$\alpha_{u}=\frac{\exp\left(\mathrm{MLP}\left(x_{v}^{u}\right)\cdot \mathrm{MLP}\left(x_{i}^{u}\right)\right)}{\sum_{k=1}^{U}\exp\left(\mathrm{MLP}\left(x_{v}^{k}\right)\cdot \mathrm{MLP}\left(x_{i}^{k}\right)\right)},$$

where MLP(·) is a multi-layer perceptron that maps a single-channel sequence into a feature vector, while $\alpha_{u}$ reflects the importance weight of the *u*-th sensor in the similarity measurement. By incorporating the weight ($\alpha_{u}$) into the calculation of the multi-channel DTW distance, we obtain Equation (22):

(22) $$d_{M}\left(X_{v},X_{i}\right)=\sum_{u=1}^{U}\alpha_{u}\cdot \min_{\pi }\sum_{(t,t^{\prime })\in \pi }\left|x_{v,t}^{u}-x_{i,t^{\prime }}^{u}\right|,$$

where this distance metric adaptively focuses on sensors sensitive to degradation while effectively mitigating noise interference.

During the system degradation process, the tail data typically best reflects the current state and the latest trends of the system. In the implementation, a fixed window of length L is used to extract the last L time points from each unlabeled sample ($X_{v}$), resulting in $X_{\tau t}$. Subsequently, a sliding window of length L is applied to the historical data ($X_{h}$) to extract all candidate segments ($\left\{X_{\tau t,j}\right\}$). Based on the Attention-DTW similarity calculation, the distance ($d_{M}\left(X_{v},X_{i}\right)$) is computed according to Equation (22), and the M most similar segments are selected as shown in Equation (23):

(23) $$X_{\tau ,v}^{i_{m}}=\underset{Y}{arg min}d\left(i,j\right),\;v,\;\;m=1,2,\ldots ,M$$

The predicted label ($y_{i_{m}}$) is derived based on Equation (24):

(24) $$y_{i_{m}}=T_{i_{m}}-e_{i_{m}}+L,\;m=1,2,\ldots ,M$$

and the final dataset is formulated as Equation (25):

(25) $$D_{v}={\left\{\left(X_{\tau ,v}^{i_{m}},y_{i_{m}}\right)\right\}}_{m=1}^{M}$$

This process builds the complete training dataset ($\left\{D_{v},Y_{v}|X_{v}\right\}$), which will be used in the subsequent CNN feature extraction network.

### 3.3. Attention-Enhanced Multi-Scale Hybrid Network for Remaining Useful Life Prediction

**Figure 5:**
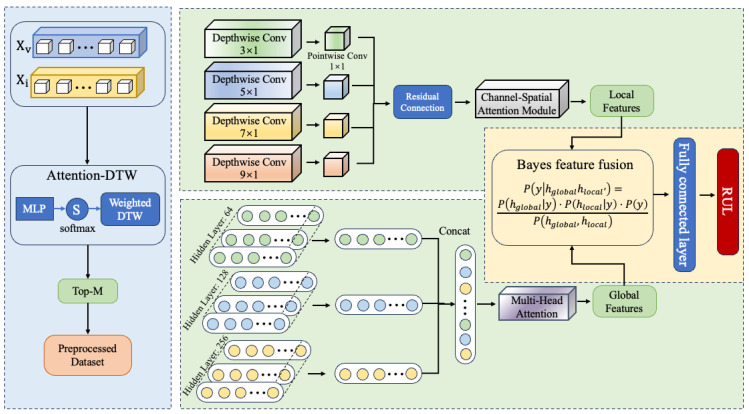
Hybrid RUL prediction network.

The extraction of features from local and long-term degradation trends in the ADAPT-RULNet structure is mainly achieved through an attention-enhanced multi-scale hybrid RUL prediction network based on Bayesian fusion, as illustrated in Figure 5.

The proposed method primarily consists of three components:

**(a) Attention-Enhanced Multi-Scale Depthwise Separable Convolution (DSC).** This module is designed to accurately identify short-term dependencies across multiple scales while reducing computational complexity. Four convolutional kernels with distinct dimensions—(3,1), (5,1), (7,1), and (9,1)—are employed to capture multi-scale features from the input data. Residual connections are introduced to preserve and enhance low-level feature information, mitigating the risk of gradient vanishing in deep networks. To further optimize feature representation, a Combined Spatial and Channel Attention Module (CSAM) is integrated, which fuses the Channel Attention Module (CAM) and the Spatial Attention Module (SAM) to dynamically weight key features and achieve efficient fusion of multi-level features.

**(b) Attention-Enhanced Multi-Scale LSTM Network.** This network is proposed to enhance the extraction of long-term degradation trends. It employs three hidden layers with dimensions of 64, 128, and 256 to capture multi-scale temporal patterns while avoiding overfitting. To improve the identification of degradation characteristics, a Multi-Head Self-Attention (MHSA) mechanism is introduced, which dynamically allocates weights to focus on the most critical time-scale features for RUL prediction. Specifically, the attention mechanism computes weights for each time-scale feature, performs weighted fusion, and integrates the feature maps extracted by the LSTM network across the three scales to generate a comprehensive global feature representation.

**(c) Bayesian Feature Fusion.** A Bayesian probability-based feature fusion approach is designed to optimally combine local and global features while mitigating the uncertainty inherent in feature extraction from different networks. The mathematical formulation is given in Equation (26):

(26) $$P\;\left(y\;|\;h_{global},h_{local}\right)=\frac{P\;\left(h_{global}\;|\;y\right)\cdot P\;\left(h_{local}\;|\;y\right)\cdot P\left(y\right)}{P\;\left(h_{global},h_{local}\right)}$$

where $P(y\;|\;h_{global},h_{local})$ represents the fused feature distribution; $P(h_{global}|y)$ and $P(h_{local}|y)$ denote the conditional probability distributions of the global and local features, respectively; and P(y) is the prior distribution.

The fused feature representation is then obtained as follows:

(27) $$h_{fused}=\underset{y}{arg max}\;P\;\left(y\;|\;h_{global},h_{local}\right)$$

Finally, the features obtained through adaptive probabilistic fusion are fed into a multi-layer fully connected network to achieve precise prediction of the equipment’s RUL. The training procedure of the Attention-Enhanced Multi-Scale Hybrid Network is presented in Algorithm 1.
**Algorithm 1** Attention-Enhanced Multi-Scale Hybrid Network Training**Require:**  1: *train_data*: Preprocessed training data  2: *train_labels*: Corresponding RUL labels  3: *val_data*: Validation data  4: *val_labels*: Corresponding RUL labels  5: *device*: Computational device (‘cpu’ or ‘cuda’)  6: *fusion_dim*: Dimension for feature fusion  7: *learning_rate*: Initial learning rate  8: *epochs*: Number of training epochs  9: *batch_size*: Batch size for training**Ensure:** 10: *trained_model*: Trained neural network model 11: **procedure** TrainNeuralNetwork(train_data, train_labels, val_data, val_labels, device, fusion_dim, learning_rate, epochs, batch_size) 12:     Initialize CNN-LSTM model with attention mechanisms 13:     Define loss function (e.g., MSE) and optimizer (e.g., AdamW) 14:     **for** epoch = 1 to epochs **do** 15:         **for** batch = 1 to len(train_data)/batch_size **do** 16:            Load batch data and labels 17:            Forward pass: compute model output 18:            Calculate loss 19:            Backward pass: compute gradients 20:            Update model parameters 21:         **end for** 22:         Validate model on validation set 23:         Compute validation loss and metrics 24:         Update learning rate scheduler if needed 25:     **end forreturn** *trained_model* 26: **end procedure**

### 3.4. Strategy for Balancing Model Complexity and Efficiency

To achieve global balancing of model complexity and predictive performance and to overcome the lack of flexibility of traditional fixed-window approaches in capturing both long-term and short-term dependencies, this study innovatively introduces the DDPG algorithm into RUL prediction. By abstracting the adaptive parameter adjustment problem of the RUL prediction model into a DRL environment, we define the state space, action space, and reward function, thereby constructing a dynamic optimization framework.

**State Space S:** The state vector of the DDPG agent is composed of the performance metrics of the current RUL prediction model and the parameters of the personalized dataset. Specifically, the state vector includes the Mean Squared Error (MSE), Akaike Information Criterion (AIC), and DTW similarity, as well as the current time-window length (L) and dataset size (M). These metrics comprehensively reflect the model’s prediction accuracy, complexity, and dataset quality. The AIC value is defined as follows:

(28) $$\mathrm{AIC}=2\phi -2\ln\left(\hat{L}\right)=2\phi +U\times L\times M\;\left[\ln(2\pi \cdot \mathrm{MSE})+1\right]$$

where ϕ denotes the total number of model parameters, *U* represents the number of sensor channels, L is the sliding-window size, M is the number of similar segments, and $\hat{L}$ is the likelihood-function value of the maximum likelihood estimation.

**Action Space A:** The agent adjusts two continuous parameters—ΔL and ΔM, which represent the adjustment magnitudes of L and M, respectively. These values are normalized within [−1,1] and mapped to the valid ranges of *L* and *M* via linear scaling, ensuring rational and feasible parameter updates.

**Reward Function R:** The reward drives the optimization process of the DDPG agent. A composite reward is designed to dynamically adjust L and M, enabling better adaptability across different degradation stages and achieving global balancing between complexity and accuracy.

*(i) Base Reward:* Negatively correlated with MSE and AIC and positively correlated with DTW similarity:

(29) $$R_{base}=-\alpha_{1}\cdot \mathrm{MSE}-\alpha_{2}\cdot \mathrm{AIC}+\alpha_{3}\cdot {\mathrm{DTW}}_{sim}$$

where $\alpha_{1},\alpha_{2}$, and $\alpha_{3}$ are weighting coefficients.

*(ii) Improvement Reward:* Measures improvement compared to the previous step:

(30) $$R_{improve}=\beta_{1}\left({\mathrm{MSE}}_{prev}-{\mathrm{MSE}}_{curr}\right)+\beta_{2}\left({\mathrm{AIC}}_{prev}-{\mathrm{AIC}}_{curr}\right)+\beta_{3}\left({\mathrm{DTW}}_{sim,curr}-{\mathrm{DTW}}_{sim,prev}\right)$$

where $\beta_{1},\beta_{2}$, and $\beta_{3}$ are weighting coefficients.

*(iii) Stability Reward:* Penalizes unstable fluctuations in recent performance:

(31) $$R_{stability}=-\gamma_{1}\cdot \mathrm{Std}\left({\mathrm{MSE}}_{recent}\right)$$

where $\gamma_{1}$ is a weighting coefficient.

*(iv) Total Reward:* The overall reward is defined as follows:

(32) $$R_{total}=R_{base}+R_{improve}+R_{stability}$$

During DDPG training, the agent iteratively interacts with the RUL prediction environment. At each time step, the actor network selects an action, and the environment updates L and M and recalculates the MSE, AIC, and DTW similarity. The corresponding reward and new state are returned, which are stored in the replay buffer. The agent periodically samples from the buffer to update actor and critic parameters, while target networks are updated via a soft-update strategy. Through iterative training, the agent progressively learns an optimal policy that adaptively adjusts L and M to balance model complexity and predictive performance. The process of DDPG for hyperparameter optimization is presented in Algorithm 2.
**Algorithm 2** DDPG for Hyperparameter Optimization**Require:**  1: *env*: Environment for RUL prediction  2: *state_dim*: Dimension of state space  3: *action_dim*: Dimension of action space  4: *action_range*: Range of actions  5: *memory_capacity*: Capacity of replay memory  6: *batch_size*: Batch size for training DDPG  7: *gamma*: Discount factor  8: *tau*: Soft update coefficient  9: *actor_lr*: Learning rate for actor network 10: *critic_lr*: Learning rate for critic network**Ensure:** 11: *ddpg_agent*: Trained DDPG agent 12: **procedure** DDPG(env, state_dim, action_dim, action_range, memory_capacity, batch_size, gamma, tau, actor_lr, critic_lr) 13:     Initialize actor network μ and critic network *Q* 14:     Initialize target networks $\mu^{\prime }$ and $Q^{\prime }$ 15:     Initialize replay memory 16:     Initialize actor and critic optimizers 17:     **for** each training step **do** 18:         Obtain current state from environment 19:         Select action using actor network 20:         Execute action in environment, obtain reward and next state 21:         Store transition in replay memory 22:         Sample random batch from replay memory 23:         Update critic network using sampled batch 24:         Update actor network using sampled batch 25:         Soft update target networks 26:     **end forreturn** *ddpg_agent* 27: **end procedure**

## 4. Experiments

### 4.1. Evaluation Metrics

The model was evaluated with the Root Mean Square Error (RMSE), accuracy, and Score function (Score). Experiments were conducted on datasets from engines and railway freight car wheels to assess the algorithm’s adaptability. Lower values of RMSE and Score indicate higher accuracy and quality of the algorithm.

(1) As a widely adopted regression metric, RMSE provides an intuitive measure of the model’s performance in predicting target values. Its definition is given by Equation (33):

(33) $$d_{i}={\hat{\mathrm{RUL}}}_{i}-{\mathrm{RUL}}_{i},\;\mathrm{RMSE}=\sqrt{\frac{1}{n}\sum_{i=1}^{n}d_{i}^{2}}$$

Here, $\hat{\mathrm{RUL}}$ and RUL are the predicted and actual RUL values. $d_{i}$ is the prediction error.

(2) Accuracy is used to evaluate the percentage of predictions within the correct range in the model’s output. If the prediction error is $e_{j}\in \left[-13,10\right]$ and $\mathrm{Cor}(e_{j})=1$, the prediction is considered reasonable; otherwise, $\mathrm{Cor}(e_{j})=0$. The mathematical formulation is provided in Equation (34):

(34) $$\mathrm{Accuracy}=\frac{100}{N}\sum_{j=1}^{N}\mathrm{Cor}\left(e_{j}\right)$$

(3) Score is a well-known RUL statistical metric and indicates better RUL estimation performance when its value is smaller. Score penalizes late RUL predictions, whereas RMSE assigns equal weights to premature and delayed predictions. Given that early fault detection holds greater significance for maintenance planning, its expression is given by Equation (35):

(35) $$\mathrm{Score}=\left\{\begin{array}{cc} \sum_{i=1}^{M}\left(e^{-\frac{d_{n}}{13}}-1\right), & \mathrm{if}\;d_{n}<0\\ \sum_{i=1}^{M}\left(e^{\frac{d_{n}}{10}}-1\right), & \mathrm{if}\;d_{n}>0 \end{array}\right.$$

where $d_{n}$ is the difference between ${\hat{\mathrm{RUL}}}_{i}$ and ${\mathrm{RUL}}_{i}$ and *M* denotes the total size of the dataset.

### 4.2. Engine Dataset Prediction

The dataset for a turbofan engine was generated by the Commercial Modular Aero-Propulsion System Simulation (C-MAPSS) platform [29]. The dataset includes four subsets, as shown in Table 1. The progression of complexity from FD001 to FD004 stems from diverse operational scenarios and failure types. Each subset is divided into training and testing components. Training components feature comprehensive run-to-failure time series from multiple engine units, while testing components present incomplete operational records preceding engine failure events. Each operational record includes 26 variables: variable 1 is the engine ID, variable 2 is the operational cycle, variables 3 to 5 are operational setting parameters that notably influence engine performance, and the remaining 21 variables are noisy sensor readings.

#### 4.2.1. Data Preprocessing

**Figure 6:**
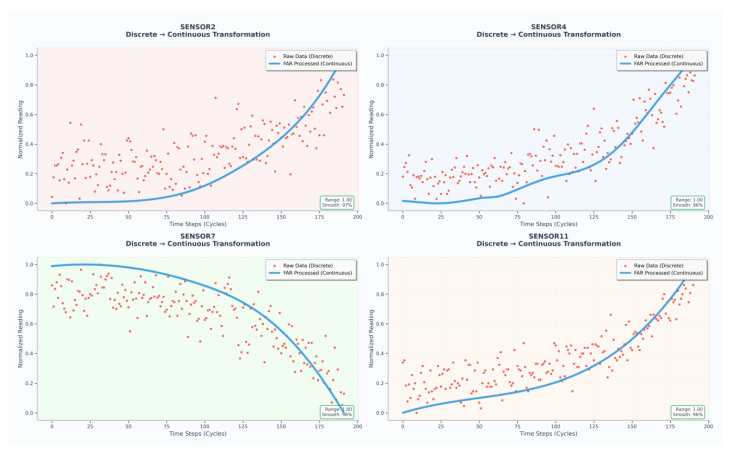
Comparative diagrams for preprocessing of sensor data.

Following the methodology proposed by Ragab et al. [30], the data was preprocessed, and fourteen sensors exhibiting degradation trends were selected, with the engine’s RUL label serving as the final input for the ADAPT-RULNet model. Taking SENSOR2, SENSOR4, SENSOR7, and SENSOR11 as representative cases, Figure 6 presents a comparative analysis between the raw sensor data and the data processed using the FAR method. The FAR technique demonstrates its efficacy by effectively eliminating high-frequency noise, mitigating localized fluctuations, and addressing inconsistencies in time-series length.

Finally, Z-score normalization was applied to all data that had undergone smoothing and interpolation processes. This step was essential to eliminate the discrepancies in scale and numerical range across different sensors, thereby accelerating the convergence of model training and avoiding dominance by high-magnitude features.

#### 4.2.2. Construction of Personalized Datasets

To achieve efficient and precise similarity measurement and sample selection, this paper proposes a strategy for personalized dataset construction with similarity degradation stages by Attention-DTW. This strategy dynamically learns the weight distribution of each sensor channel, enabling the DTW alignment process to adaptively focus on key modal information that is more indicative of degradation trends.

**Figure 7:**
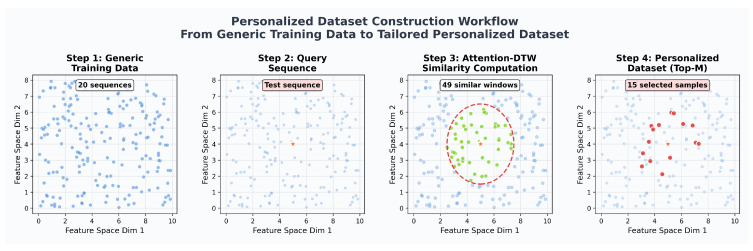
The construction process of the personalized datasets.

As illustrated in Figure 7, the construction process of the personalized dataset includes the following steps: Step 1—raw sequences are extracted from the general training set and mapped to a two-dimensional feature space for visualization; Step 2—a test sequence of length M is input as the query object; Step 3—the candidate windows most similar to the test sequence are screened from the training set through Attention-DTW similarity calculation; Step —the top M samples are selected based on similarity ranking to form the personalized dataset, thereby providing a more reliable data foundation for degradation trend analysis.

#### 4.2.3. Parameter Selection

The parameter configuration of the proposed ADAPT-RULNet model is listed in Table 2.

For model optimization, the AdamW optimizer is employed to update the parameters, initialized with a learning rate of 0.001. To further enhance the training performance, the learning rate is dynamically adjusted using the Cosine Annealing Warm Restarts scheduler, where the initial cycle ($T_{0}$) is set to 10, the cycle multiplier ($T_{\mathrm{mult}}$) is set to 2, and the minimum learning rate is $1\times 10^{-6}$. Additionally, early stopping prevents overfitting by terminating training when validation loss plateaus for 20 epochs.

**Figure 8:**
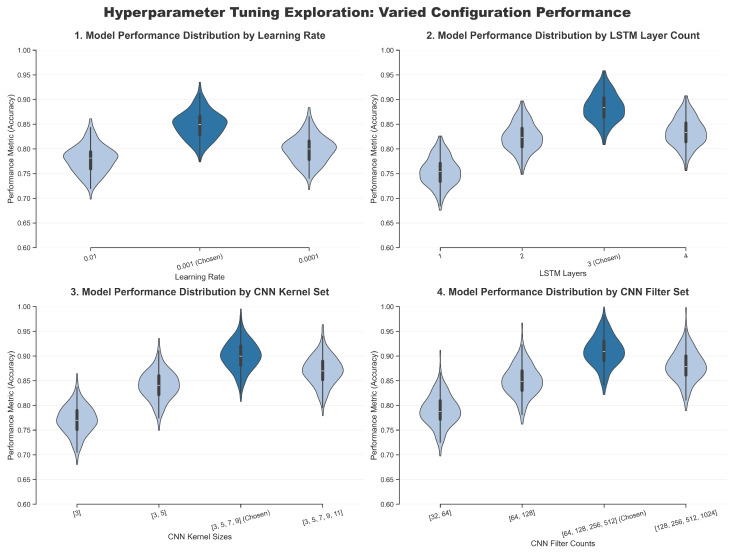
Comparison of accuracy of different parameters.

During hyperparameter selection, comparative experiments were conducted on the learning rate, the number of LSTM layers, the kernel sizes of the CNN, and the number of filters. Multiple experiments were performed to evaluate their impact on RUL prediction accuracy, and the most suitable hyperparameters were identified accordingly. The comparative results are shown in Figure 8, where the violin-shaped regions represent kernel density estimates, the black lines indicate the upper and lower bounds after removing outliers, the horizontal lines denote the medians, and the white dots represent outliers. As illustrated in the figure, the highest prediction accuracy is achieved when the learning rate is 0.001, the number of LSTM layers is 3, the CNN kernel sizes are [3, 5, 7, 9], and the CNN filter counts are [64, 128, 256, 512].

#### 4.2.4. Reinforcement Learning-Driven Adaptive Parameter Tuning

The parameter adjustment problem is formulated as a sequential decision-making task within the DDPG framework, which adaptively optimizes the key parameters in personalized dataset construction through interaction with the prediction environment to learn the most effective policy. Specifically, the state space of the agent is designed as a five-dimensional vector comprising the performance metrics of the current RUL estimation model and the parameters of the personalized datasets, including the window length (L), the dataset size (M), MSE, AIC value, and DTW similarity.

**Figure 9:**
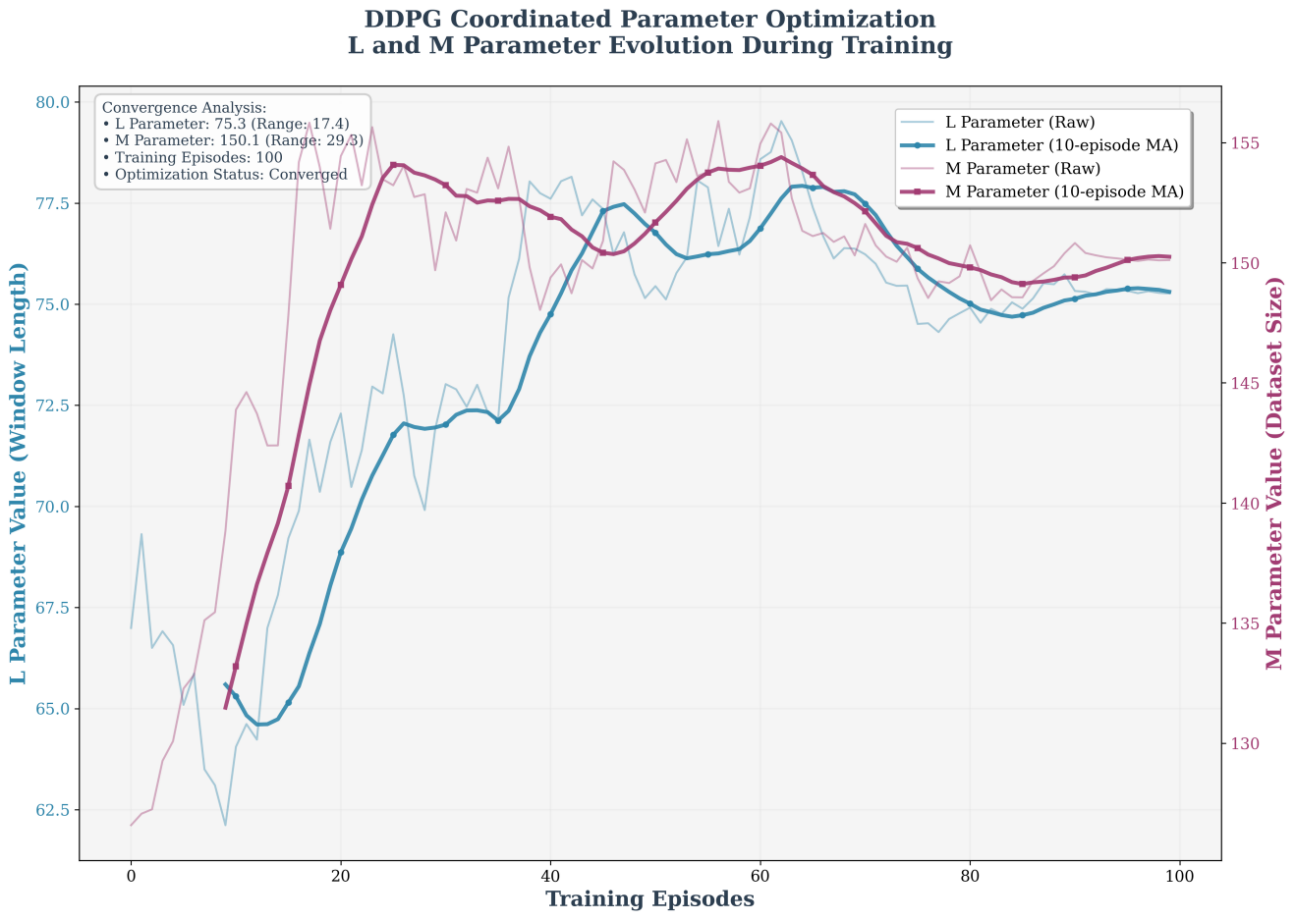
The evolution and convergence analysis of the L and M parameters during DDPG training.

The RUL prediction environment is responsible for updating the L and M parameters based on the actions of the DDPG agent and providing corresponding performance feedback. Figure 9 illustrates the evolution trends of the L and M parameters during DDPG training. The horizontal axis denotes the training episodes, the left vertical axis represents L, and the right vertical axis represents M. The light-blue and dark-blue curves represent the raw values and the 10-episode moving average of L, respectively, while the light-red and dark-red curves represent the raw values and the 10-episode moving average of M, respectively. Moving average processing provides a clearer depiction of the overall trend of parameter changes.

The convergence analysis in the upper-left corner indicates that L ultimately converges to 75.3 and M converges to 150.1, with the optimization state marked as “Converged”. During initial training, the raw values of L and M exhibit significant fluctuations, reflecting the active exploration of parameter adjustments. As training progresses, the moving-average curves gradually smooth out and stabilize in the mid-to-late stages, validating the effective optimization and convergence process of the DDPG algorithm.

**Figure 10:**
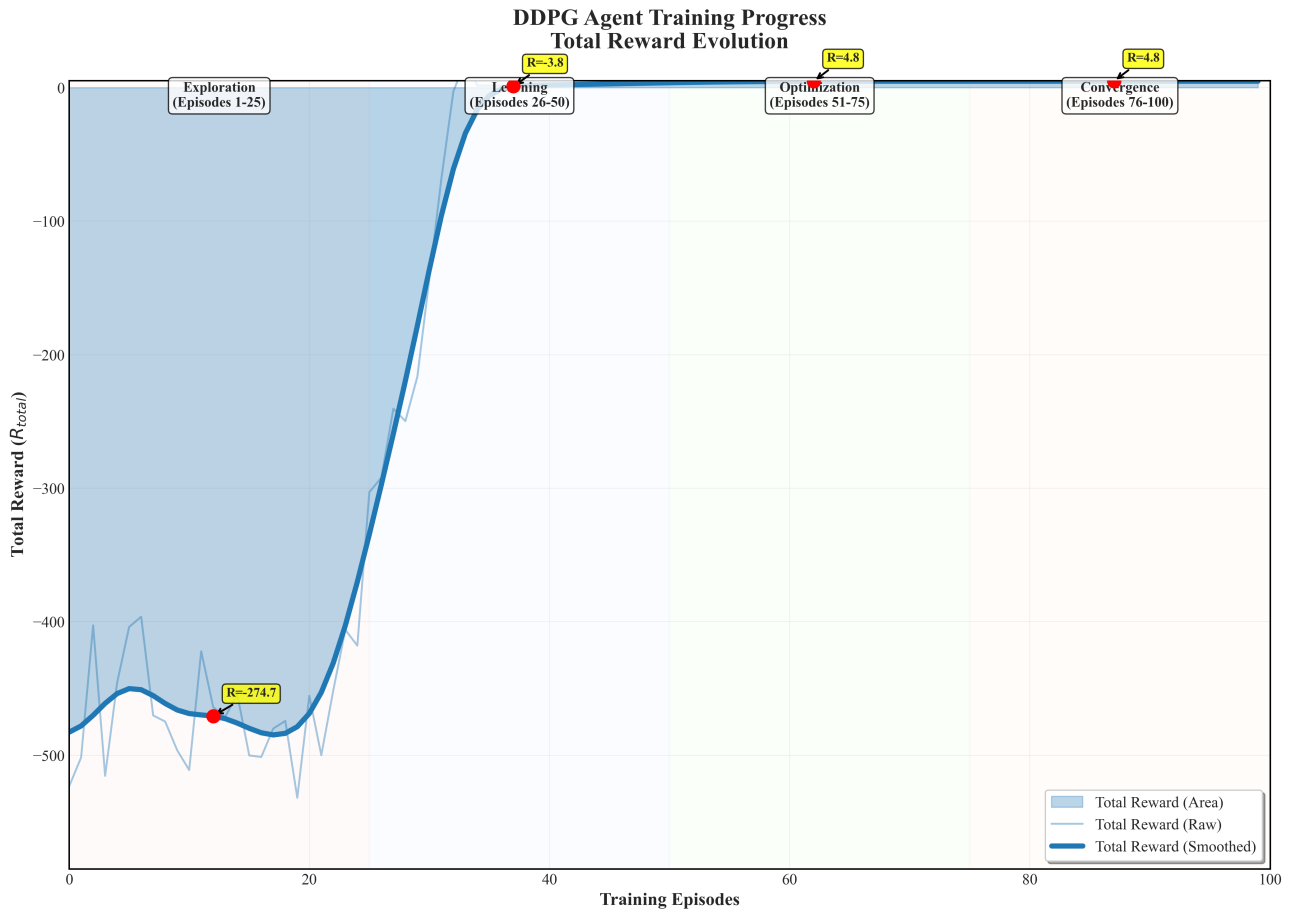
Evolution of total reward.

Figure 10 illustrates the evolution of the total reward of the RUL prediction model based on the DDPG algorithm over 100 training episodes. As shown in the figure, the agent’s learning process undergoes four distinct stages: an initial exploration phase characterized by low and highly fluctuating rewards; a subsequent learning and optimization phase with gradually increasing rewards; and, finally, a convergence phase where stable, high-performance rewards are achieved. This evolutionary trend confirms that the DDPG algorithm can effectively learn the optimal strategy for adaptively adjusting the time-window length (L) and the dataset size (M), thereby achieving a dynamic balance between model complexity and prediction accuracy.

**Figure 11:**
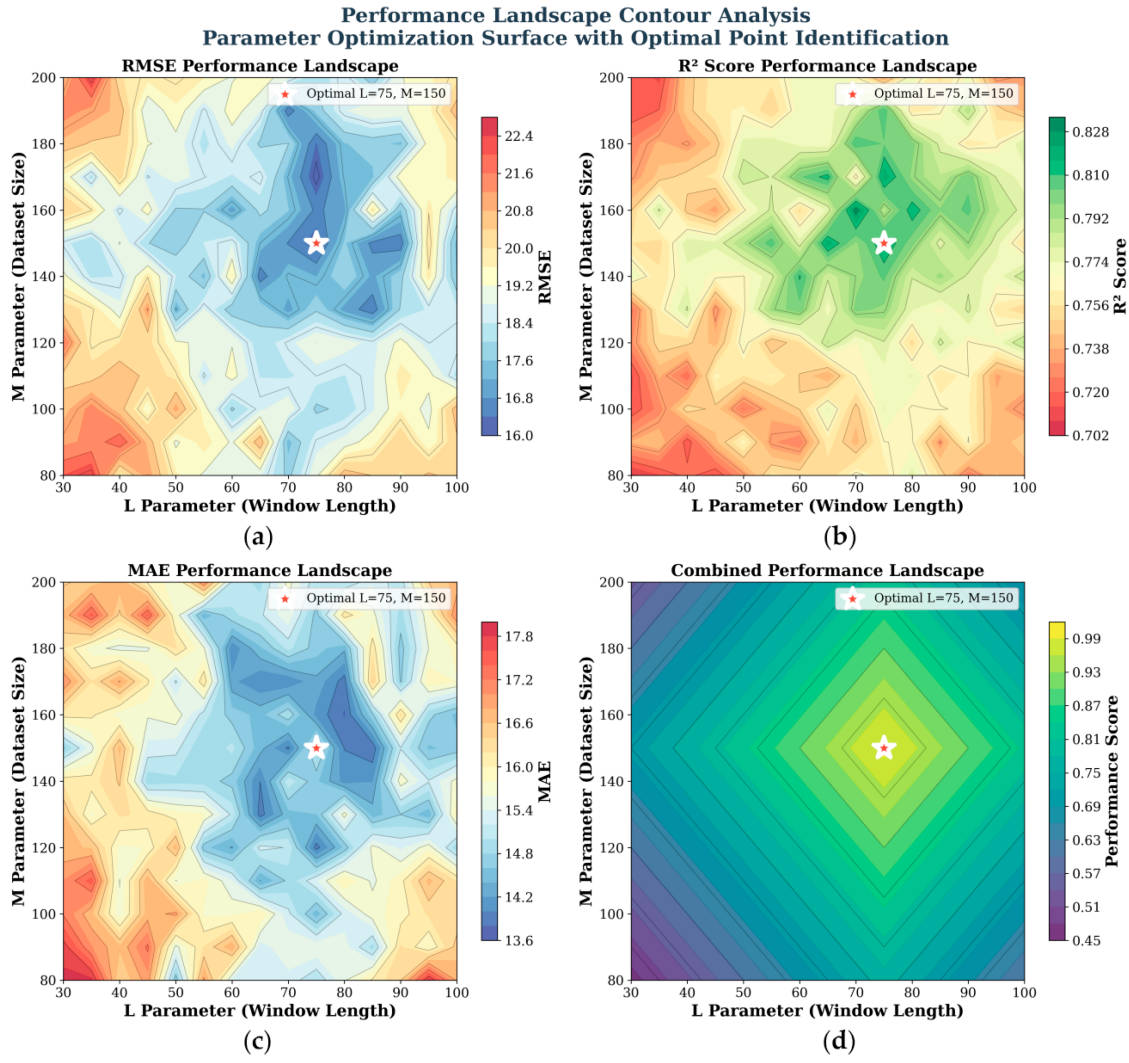
Contour analysis of model performance distribution with respect to the L and M parameters. Subplots show results for (**a**) RMSE, (**b**) $R^{2}$ Score, (**c**) MAE, and (**d**) comprehensive performance. The white star denotes the optimal parameter point (L=75, M=150).

Figure 11 presents a contour analysis of model performance distribution based on the combinations of L and M, including four subplots for RMSE, $R^{2}$ Score, MAE, and comprehensive performance. In each subplot, the values of L are plotted along the horizontal axis, whereas the values of M are plotted along the vertical axis. The color gradient and contour lines visualize the performance under different parameter combinations, where cool tones (e.g., blue) indicate better performance and warm tones (e.g., red) indicate poorer performance.

Each subplot marks the optimal parameter point as “Optimal L=75 and M=150” (denoted by a white star), demonstrating that this combination achieves optimal performance in terms of RMSE (measuring the magnitude of prediction error, where lower values are better), $R^{2}$ Score (reflecting the model’s ability to explain data variation, where values closer to 1 are better), MAE (calculating the mean absolute error between predictions and true values, where lower values are better), and comprehensive performance. Such visualizations intuitively illustrate the impact of parameter interactions on model performance, identify universally optimal parameters, and provide critical insights for parameter tuning and model performance validation, thereby aiding in understanding parameter sensitivity regions and optimization stability.

#### 4.2.5. Experimental Results

This section presents the experimental results of the proposed adaptive RUL prediction framework on the C-MAPSS dataset and compares it with current mainstream approaches both quantitatively and qualitatively, in order to validate the effectiveness of the proposed method.

**Figure 12:**
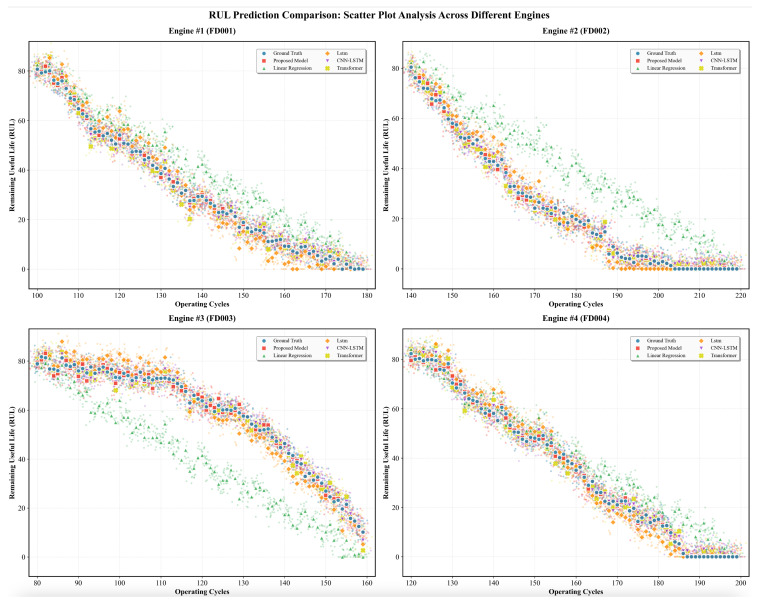
Prediction results of ADAPT-RULNet on the C-MAPSS dataset.

The prediction results of ADAPT-RULNet on the C-MAPSS dataset are shown in Figure 12. The ADAPT-RULNet model demonstrates a better alignment between predicted and observed RUL curves across different subsets of the dataset. Notably, as the actual RUL diminishes—indicating the engine’s approach to failure—the prediction error is markedly reduced, with the majority of the predicted values falling within a reasonable error margin. This indicates the model’s proficiency in accurately identifying significant degradation features during the latter stages of equipment deterioration, particularly within the pivotal prediction window, thereby facilitating precise RUL forecasting.

For the purpose of evaluating the performance of the proposed framework, we further compared ADAPT-RULNet with recent prediction methods. The comparative results are summarized in Table 3.

**Figure 13:**
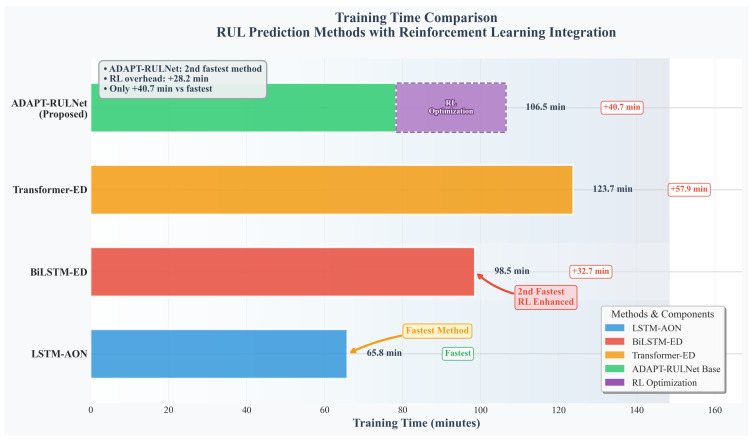
Comparison of computational performance of algorithms.

From a horizontal perspective, the evaluation metrics (RMSE, Score, and Accuracy) show a gradual decline across the FD001 to FD004 datasets, reflecting the increased complexity of the dataset under the diversity of operating conditions and fault modes, which brings greater challenges to the prediction task. In a vertical comparative analysis, the ADAPT-RULNet model proposed in this study demonstrates notable competitiveness across all evaluation metrics. Its average RMSE and average Accuracy achieve the best optimal values among existing methods, clearly indicating that the proposed framework surpasses previous solutions with regard to overall prediction accuracy. Furthermore, we investigated the computational performance of the models. As shown in Figure 13, by comparing the computational efficiency of advanced models listed in Table 3, including ADAPT-RULNet, Transformer-ED, BiLSTM-ED, BiGRU-AS, and LSTM-AON, we observed that the proposed method achieves an upper–middle level of efficiency. In addition, further analysis of the neural network component and the reinforcement learning training cost revealed that the efficiency bottleneck of the proposed method primarily lies in the reinforcement learning module. In future work, lightweight architectures and meta-learning strategies will be explored to further enhance the efficiency of the reinforcement learning component. Although further performance improvements might be attainable through a more extensive hyperparameter search and more efficient and accurate data preprocessing, the proposed and validated RUL prediction framework, which integrates attention mechanisms and DRL, leverages its adaptive advantages to effectively address the inherent limitations of traditional deep learning methods in handling heterogeneous sensor data and complex operating condition failure patterns. This not only provides an effective approach to reducing prediction inaccuracies but also offers practical references for researchers in hyperparameter optimization.

### 4.3. Prediction of Railway Freight Car Wheels Dataset

To additionally confirm the generalization capability of the ADAPT-RULNet framework in engineering practice, we migrated it to the non-public TWDS railway freight car wheel dimension monitoring dataset under real-world railway operation conditions for experimental verification.

(1) **Freight Car Wheel Dataset**

The wheel dataset comprises 400 sets of wheel inspection data, each with an operational cycle exceeding two years, as detailed in [13]. The dataset is structured with 17 fields, among which fields C1 to C6 contain wheel positioning information utilized for the precise identification and tracking of each wheel’s location. The actual deterioration information is documented in fields C7 to C16, which meticulously reflect the wear and performance evolution of the wheels under actual operating conditions.

(2) **Data preprocessing**

Because of the effects of wheel vibration and sensor data acquisition errors, the raw data exhibit significant fluctuations. Additionally, during wireless transmission, network latency and external environmental interference can cause data loss, noise, and anomalies.

**Figure 14:**
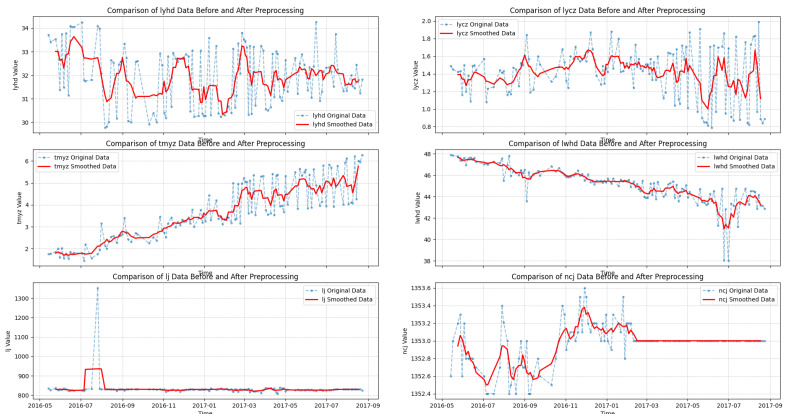
Data trend after preprocessing.

Therefore, it is necessary to perform Functional Adaptive Regression (FAR) initialization on the raw data. Subsequently, six sensors that reflect the wear evolution patterns were identified, including left wheel thickness (lyhd), left wheel tread-wear depth (lycz), rim wear (tmyz), wheel-axle diameter parameter (lwhd), mileage data (lj), and inner measurement value (ncj). These parameters comprehensively characterize the wear evolution of wheel-axle components under actual operating conditions. The preprocessing results of the wheel dataset are illustrated in Figure 14, which shows that FAR effectively eliminates high-frequency noise, mitigates localized fluctuations, and enhances the consistency of time-series signals.

**Figure 15:**
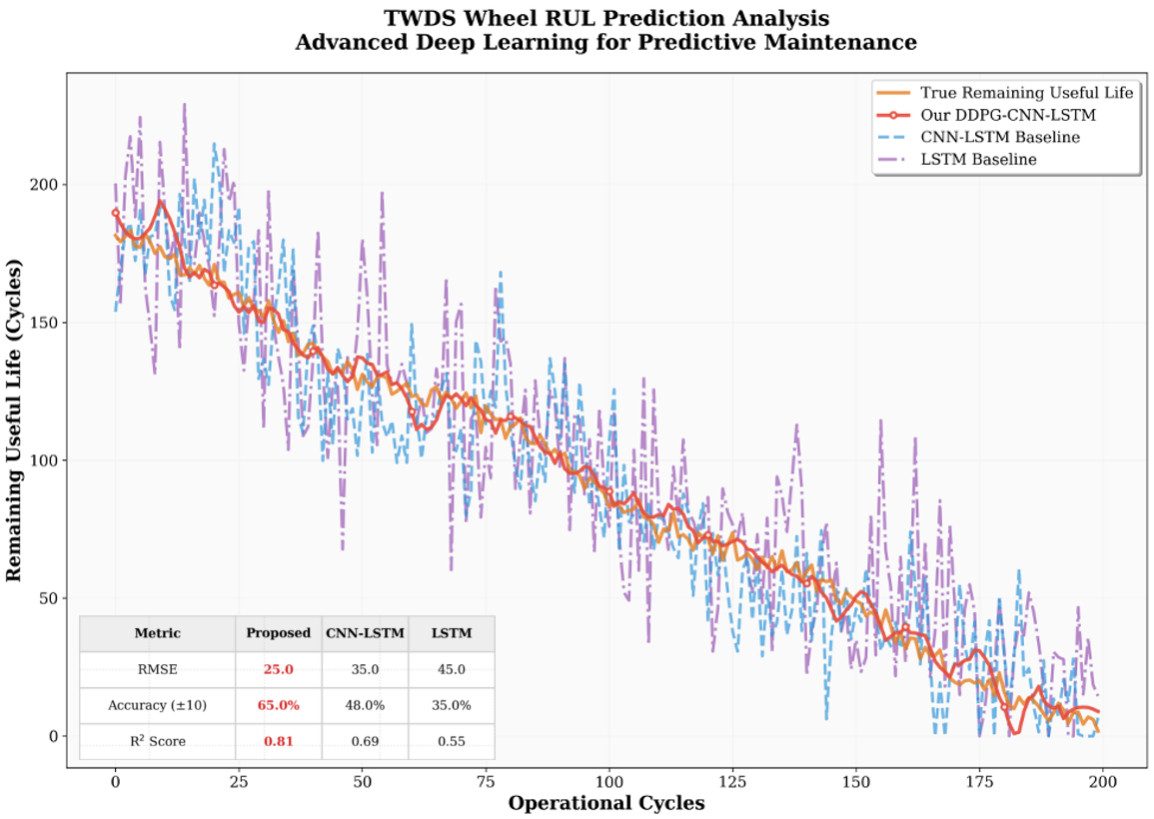
The RUL prediction results of ADAPT-RULNet on the TWDS dataset.

The pre-trained weights of the ADAPT-RULNet model were used on the railway freight car wheel dataset for RUL prediction. Experimental results presented in Figure 15 demonstrate that the proposed model achieves stable predictive performance in the quantitative analysis on the TWDS dataset in comparison with the baseline LSTM and CNN-LSTM models. As a result of heterogeneous data sources and operational contexts, its absolute performance metrics exhibit some differences relative to its performance on the C-MAPSS dataset. However, the model demonstrates better transfer learning capabilities, effectively capturing degradation trends in non-public data. Specifically, on the TWDS dataset, ADAPT-RULNet maintains an average RMSE within an acceptable range, and its prediction curves exhibit high consistency with the actual RUL trends. These findings further validate the practical value of our proposed framework in handling unknown or complex real-world data, laying the foundation for future industrial deployment.

## 5. Conclusions

Accurate prediction of RUL is of paramount importance in optimizing maintenance strategies, reducing operational expenses, and ensuring the operational safety of equipment. This study introduces an adaptive framework for RUL prediction that integrates attention mechanisms and RL. The primary objective is to address challenges related to the adaptability of the prediction process, the accuracy of prediction results, and the generalization ability of the prediction model. Additionally, the method employs the FAR approach for data preprocessing and utilizes attention mechanism-based DTW to construct personalized datasets, ensuring high-quality signal input while improving the efficiency of feature extraction. The proposed attention-enhanced CNN-LSTM hybrid network architecture achieves the fusion of local temporal and global dependency features, enhancing the accuracy of RUL prediction, particularly providing high-precision predictions during the later stages of equipment degradation. Finally, to balance model complexity and prediction performance, parameter tuning is transformed into an MDP model, and techniques including experience replay and target-network soft updates of the DDPG algorithm are adopted to adaptively adjust key parameters in personalized dataset construction. The method’s efficacy was validated on datasets covering two distinct components. Comparative experiments with various current DL methods were conducted. The findings indicate that the new approach achieves the highest accuracy and average metrics.

However, our proposed method also has limitations, especially in terms of model performance and interpretability. In upcoming research, we plan to continuously optimize hyperparameters and the reinforcement learning reward function, as well as further improve the computational efficiency of the algorithm. In addition, we will explore physics-informed deep learning by integrating physical models with neural networks to gain deeper insights into the degradation mechanisms of mechanical components, thereby enhancing both interpretability and predictive accuracy. We also aim to incorporate Bayesian neural networks for uncertainty estimation, providing more reliable confidence intervals for RUL predictions and enabling decision-makers to assess risks more scientifically. Furthermore, the integration of the RUL prediction model with digital twin technology will be pursued to achieve real-time monitoring and online updating of equipment status, significantly improving prediction accuracy and timeliness. Through these forward-looking studies, we expect to advance the field of RUL prediction and provide stronger technical support for equipment maintenance and operational management. In future work, we plan to continuously optimize hyperparameters and the reinforcement learning reward function, as well as further improve the computational efficiency of the algorithm. In addition, we will explore physics-informed deep learning by integrating physical models with neural networks to gain deeper insights into the degradation mechanisms of mechanical components, thereby enhancing both interpretability and predictive accuracy. We also aim to incorporate Bayesian neural networks for uncertainty estimation, providing more reliable confidence intervals for RUL predictions and enabling decision-makers to assess risks more scientifically. Furthermore, the integration of the RUL prediction model with digital-twin technology will be pursued to achieve real-time monitoring and online updating of equipment status, significantly improving prediction accuracy and timeliness. Through these forward-looking studies, we expect to advance the field of RUL prediction and provide stronger technical support for equipment maintenance and operational management.

## Figures and Tables

**Table 1:** Overview of the C-MAPSS datasets.

Dataset	FD001	FD002	FD003	FD004
Training Units	100	260	100	249
Testing Units	100	259	100	248
Operating Conditions (OCs)	1	6	1	6
Fault Modes (FMs)	1	1	2	2

**Table 2:** The parameters of the ADAPT-RULNet model.

Parameter	Value
input_dim	14
cnn_filters	[64, 128, 256, 512]
cnn_kernels	[3, 5, 7, 9]
lstm_hidden	256
lstm_layers	3
fusion_dim	512
Learning rate	0.001
Training epochs	200
Window length	50
Loss function	NSE Loss
Batch size	64
Optimizer	AdamW

**Table 3:** Comparison of evaluation metrics.

Method	Metric	FD001	FD002	FD003	FD004	Average
LSTM-AON [31]	Score	284	2454	428	4708	1968.5
	Accuracy	64	62	57	34	54.25
	RMSE	13.68	20.81	**15.53**	27.31	19.332
BiLSTM-ED [32]	Score	273	3099	574	3202	1787
	Accuracy	57	49	42	40	47
	RMSE	14.74	22.07	17.48	23.49	19.445
KGHM [33]	Score	250.99	1131.03	333.44	3356.10	**1267.89**
	Accuracy	67	46	59	45	54.25
	RMSE	13.18	13.25	13.54	19.86	14.958
CNN-LSTM [34]	Score	256	–	–	–	–
	Accuracy	58	–	–	–	–
	RMSE	13.34	–	–	–	–
Attention CNN-RUL [35]	Score	217.02	789.32	216.79	1107.96	582.77
	Accuracy	59	–	–	–	–
	RMSE	10.43	11.02	10.03	16.23	11.93
Transformer-ED [36]	Score	286	2799	574	4655	2078.5
	Accuracy	64	61	57	44	56.50
	RMSE	13.87	20.76	17.42	24.41	**19.115**
**ADAPT-RULNet**	Score	275	2324	460	3155	1553.5
	Accuracy	**68**	**65**	**60**	**47**	**60.0**
	RMSE	12.48	**19.45**	**14.92**	**22.97**	**17.45**

## Data Availability

The original contributions presented in this study are included in the article. Further inquiries can be directed to the corresponding author.
